# NLRP3 Inflammasome and Its Central Role in the Cardiovascular Diseases

**DOI:** 10.1155/2020/4293206

**Published:** 2020-04-14

**Authors:** Yeqing Tong, Zhihong Wang, Li Cai, Liangqiang Lin, Jiafa Liu, Jinquan Cheng

**Affiliations:** ^1^Center for Disease Control and Prevention, 430079 Hubei, China; ^2^Key Laboratory of Molecular Biology of Guangdong Province, Center for Disease Control and Prevention, Shenzhen 518055, China; ^3^Department of Neurology, Shenzhen NO. 2 People's Hospital, The First Affiliated Hospital of Shenzhen University, Shenzhen 518035, China; ^4^Wuhan Center for Disease Control and Prevention, Wuhan 430015, China; ^5^School of Health Sciences, Wuhan University, Wuhan 430071, China; ^6^School of Public Health and Primary Care, The Chinese University of Hong Kong, Shatin Hong Kong, China

## Abstract

**Materials:**

The NLRP3 inflammasome controls the activation of the proteolytic enzyme caspase-1. Caspase-1 in turn regulates the maturation of the proinflammasome cytokines IL-1*β* and IL-18, which leads to an inflammatory response. We made a mini-review on the association of regulatory mechanisms of NLRP3 inflammasome with the development of cardiovascular diseases systematically based on the recent research studies. *Discussion*. The inflammasome plays an indispensable role in the development of atherosclerosis, coronary heart diseases (CHD), and heart ischemia-reperfusion (I/R) injury, and NLRP3 inflammasome may become a new target for the prevention and treatment of cardiovascular diseases. Effective regulation of NLRP3 may help prevent or even treat cardiovascular diseases.

**Conclusion:**

This mini-review focuses on the association of regulatory mechanisms of NLRP3 inflammasome with the development of cardiovascular diseases, which may supply some important clues for future therapies and novel drug targets for cardiovascular diseases.

## 1. Introduction

The inflammasome, a multiprotein complex macromolecular intracellular protein which supplies the platform for promoting the maturation of inflammatory cytokines, could promote the maturation of inflammatory cytokines, such as IL-1*β* and IL-18 [1–5]. These cytokines are extremely powerful molecules with myriad functions that are widely and rapidly induced in the cardiovascular diseases upon infection, trauma, or stress. Therefore, inflammasome is likely to control the inflammation in the development of cardiovascular diseases [6–8]. NLRP3 inflammasome, the most typically inflammasome which could be activated by crystal or particle pathogen damage-associated molecular patterns (PAMPs) and ischemic hypoxia danger-associated molecular patterns (DAMPs), can promote the secretion of IL-1*β* and IL-18 [9–11]. Through these mechanisms, it promotes atherosclerosis (AS), coronary heart diseases (CHD), heart ischemia-reperfusion (I/R) injury, and so on [12]. Thus, NLRP3 inflammasome may play a critical role in the cardiovascular diseases physiopathology and act as a proinflammatory mediator; it has become the focus of researchers in recent years. Researches on the role of NLRP3 inflammasome in the cardiovascular diseases are on the focus stage and have made a lot of great progress. However, a number of questions deserve further investigation. How the NLRP3 inflammasome is involved in other cardiovascular diseases, such as hypertension, arrhythmia, and heart failure, remains not very clear. In addition, the exact molecular mechanisms by which NLRP3 inflammasome is activated should also be further examined, too. Whether this complex protein is biochemically and genetically regulated or not may be a new focus in the coming years. Clinical trials have confirmed that IL-1*β* and its receptor antagonist could be used to treat a variety of cardiovascular diseases [13, 14], and the widely used drug glyburide played a crucial role in the treatment of cardiovascular diseases through the inhibition of the NLRP3 inflammasome [15]. Thus, investigations into NLRP3 inflammasome will shed light on the pathogenesis of cardiovascular diseases and provide critical clues for seeking new targets for clinical cardiovascular diseases drug development.

Despite the potential significance of NLRP3 inflammasome in the pathogenesis of several diseases, emerging evidence suggests that NLRP3 inflammasome events are associated with cardiovascular diseases conditions. Details on the activation mechanism of the NLRP3 inflammasome by a variety of stimulators have yet to be systematic reported [16]. In view of its importance and value in cardiovascular diseases, we systematically reviewed the recent research advances in NLRP3 inflammasome, particularly its specialized role in the cardiovascular diseases. In this review, we summarized the role of NLRP3 in inflammatory response and discussed the relationship between NLRP3 and cardiovascular diseases. We also provided insights into new treatment strategies for targeting NLRP3 inflammasome, as well as the upstream and downstream components of NLRP3 in alleviating cardiovascular diseases.

## 2. Structure of NLRP3 Inflammasome

**Figure 1:**
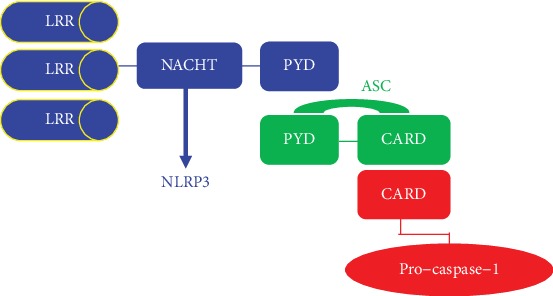
Structure of NLRP3 inflammasome.

NLRP3, the main component of the NLRP3 inflammasome which consists of N-terminal and C-terminal function structural domain, was known as a novel inflammatory gene [13, 17, 18] .The structure of NLRP3 inflammasome is described in Figure 1.

The N-terminal domain includes the hot protein pyrin domain (PYD), the caspase-associated recruitment domain (CARD), and the nucleotide-binding oligomerization domain (NOD/NACHT); the C-terminal domain includes the leucine-rich repeat (LRR) which provides a bracket to identify pathogen-associated patterns and other ligands. When ligands are identified by LRR, the NOD structure domain rearranges and triggers its biological effects [13, 19].

NLRP3 inflammasome, a new inflammasome which oligomerizes upon activation, is constituted by NLRP3, ASC, and pro-caspase-1 [20]. First and foremost, its activation will result in the recruitment of ASC through homotypic PYD-PYD interactions. Secondly, ASC forms large speck-like structures and recruits pro-caspase-1 via CARD-CARD contact, leading to the autocatalytic activation of caspase-1 [21]. Finally, activated caspase-1 converts the inactive pro-IL-1*β* and pro-IL-18 into their activated and secreted forms, mediating the subsequent responses.

## 3. Mechanisms of NLRP3 Inflammasome Activation

**Figure 2:**
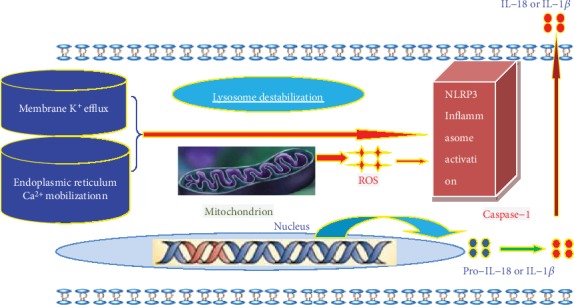
Mechanisms of NLRP3 inflammasome activation.

NLRP3 inflammasome is assembled and activated in certain classical types of mechanisms such as the lysosome destabilization, the K^+^ efflux, and Ca^2+^ mobilization as well as the ROS; the mechanisms of NLRP3 Inflammasome activation are described in Figure 2.

### 3.1. The Lysosome Destabilization Mediating Activation Pathway

The activated pathway mediated by the lysosome destabilization is mainly to activate caspase-1 to process the proinflammatory cytokines interleukin- (IL-) 1*β* and IL-18. The studies found that urea, cholesterol crystal, and aseptic materials were swallowed into the intracellular to destroy the stability of lysosome membrane and then activate the lysosomal proteases and caspase-1, further activate NLRP3 inflammasome, and promote the process of proinflammatory cytokines interleukin- (IL-) 1*β* and IL-18 damaging the body [22].

### 3.2. The K^+^ Efflux- and Ca^2+^ Mobilization-Mediated Activation Pathway

The K^+^ efflux- and Ca^2+^ mobilization-mediated pathway may play a critical role in triggering the NLRP3 inflammasome activation. It can be activated by two pathways: (1) The purinergic 2X7 receptor (P2X7R) is in the upstream of NLRP3 activation. The extracellular ATP is involved in the formation of P2X7R which triggers the K^+^ efflux. K^+^ efflux results in low K^+^ concentrations in the intracellular environment, leading to mitochondrial dysfunction, apoptosis, and the subsequent release of ROS and oxidative mtDNA, which can activate the NLRP3 inflammasome [23, 24]. (2) In response to ATP and other stimuli, Ca^2+^ released from endoplasmic reticulum storage or the extracellular space can trigger mitochondrial damage, which can also activate NLRP3 inflammasome [25].

### 3.3. The ROS-Mediated Activation Pathway

Reactive oxygen species (ROS), a powerful oxidant which is mainly produced by the mitochondria, could trigger oxidative stress and activate NLRP3 inflammasome [26]. The complex of thioredoxin and thioredoxin-interacting protein (TXNIP) could dissociate in the high ROS level circumstance. The subsequent binding of TXNIP and NLRP3 leads to the activation of TXNIP-NLRP3 inflammasome and recruitment of ASC and pro-caspase-1 as well as the formation of the active inflammasome complex [27]. Studies have found that reducing the damage of mitochondria by regulating mitochondrial autophagy could inhibit ROS from inducing NLRP3 inflammasome activation. Absence of autophagy will increase the activation of the NLRP3 inflammasome dramatically.

Mitochondrial dysfunction acts in the upstream of NLRP3 activation by providing ROS to trigger NLRP3 oligomerization or by inducing *α*-tubulin acetylation to relocate mitochondria to the proximity of NLRP3 [28]. In addition, mitochondria work as a platform for inflammasome assembly. Mitochondrial function may also depend on the downstream of NLRP3 activation. While the molecular mechanisms of mitochondrial dysfunction associated with NLRP3 activation are still unclear, they might be involved in the perturbation of mitochondria by K^+^ efflux and subsequent intracellular disequilibrium [29]. Thus, mitochondria ROS and NLRP3 machinery appear to be closely interwoven at multiple levels.

## 4. The Role of NLRP3 Inflammasomes in the Cardiovascular Diseases

Activation of the NLRP3 inflammasome by these mechanisms has been discovered in various disorders, including metabolic syndrome, type 2 diabetes, atherosclerosis, gout, reperfusion injury of the heart, neurodegeneration, such as Alzheimer's disease, chronic kidney diseases, and more, and more studies suggest that NLRP3 inflammasome is involved in the development of cardiovascular diseases.

### 4.1. The Association between NLRP3 Inflammasome and Coronary Heart Diseases (CHD)

**Figure 3:**
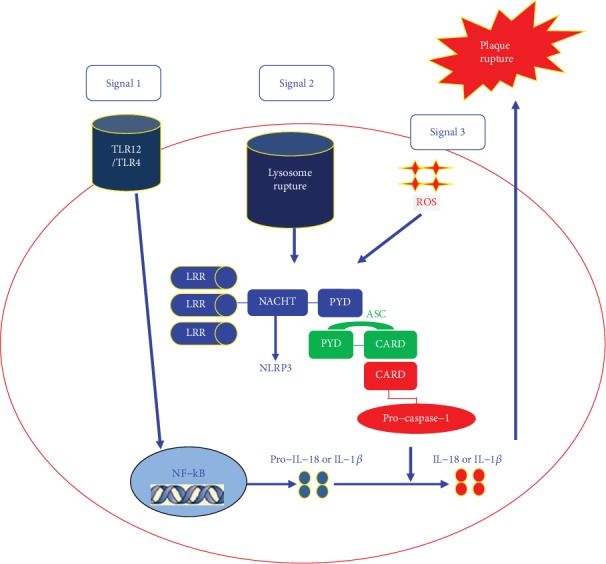
The association between NLRP3 inflammasomes and coronary heart diseases (CHD) in macrophages.

NLRP3 plays a very important role in the early stage of CHD. Low-density lipoprotein (LDL) promotes a cholesterol crystal to deposit in the vessel wall. Then, the macrophages phagocytize the lipoprotein and turn themselves into foam cells. Foam cells are activated by the following mechanisms to initiate inflammatory cycle reaction: (1) The macrophages phagocytize lysosome and then lysosomes are damaged and release ROS and protease to activate NLRP3 [30, 31]. (2) The TLR- (Toll-like receptor-) 12/TLR-4 located in the capsular identifies minimally oxidized LDL and free fatty acids and raises the myeloid differentiation primary response gene 88 and interferon TIR domain-containing adapter-inducing interferon beta (TRIF) to induce nuclear factor-kappa B (NF-*κ*B). NF-*κ*B promotes intracellular NLRP3 gene and IL-l*β* precursor expression to promote inflammation [32]. (3) The proinflammatory factors induce macrophage, neutrophil, lymphocyte, vascular smooth muscle cell infiltration and activation causing cell death and the accumulation of extracellular cholesterol and cellulose and promoting calcium phosphate crystallization deposition. The deposited crystallization calcium further breaks the lysosome of macrophages [33]. (4) The IL-l*β* raises mononuclear cells to activate platelets and promotes the release of themselves [34, 35]. (5) The activated macrophages can generate IL-18 causing more vascular smooth muscle cell necrosis and releasing the organization metalloproteinases to reduce the stability of the plaques [36]. The above mechanisms form the cycle reaction could make plaque size more big and plaque stability more serious. The detailed association between NLRP3 inflammasome and CHD is described in Figure 3.

### 4.2. The Association between NLRP3 Inflammasome and Myocardial Ischemia/Reperfusion (I/R) Injury

**Figure 4:**
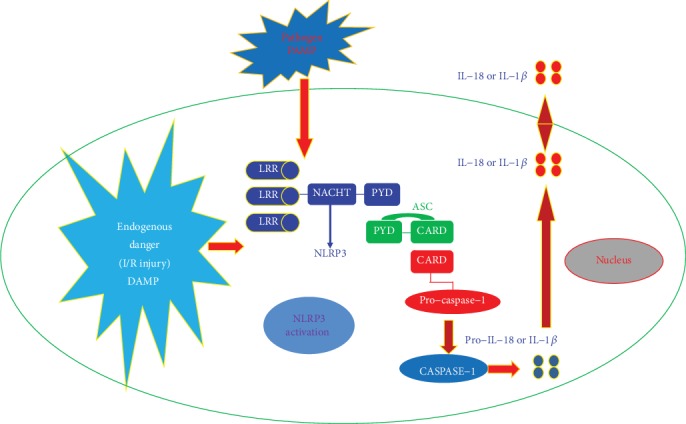
The association between NLRP3 inflammasome and myocardial ischemia/reperfusion (I/R) injury in cardiomyocytes.

Inflammation plays a key role in the pathophysiology of the I/R injury [37, 38]; however, the mechanism how myocardial I/R induces inflammation remains unclear. Recent evidence indicates that a sterile inflammatory response triggered by tissue damage is mediated through a multiple-protein complex called the NLRP3 inflammasome. Inflammatory response is initiated by the detection of PAMPs and/or DAMPs via extracellular and intracellular pattern recognition receptors [39, 40]. The inflammasome is an initial sensor for danger signals in myocardial I/R injury. Kawaguchi et al. have found that inflammasome activation in cardiac fibroblasts was crucially involved in the initial inflammatory response after myocardial I/R injury [41]. NLRP3 inflammasome was formed by I/R, and its subsequent activation of inflammasomes led to IL-1*β* production, resulting in inflammatory responses such as inflammatory cell infiltration and cytokine expression in the heart [42]. The activated NLRP3 inflammasome could integrate ASC to activate caspase-1. In mice deficient in apoptosis-associated speck-like adaptor protein and caspase-1, these inflammatory responses and subsequent injuries, including infarct development, myocardial fibrosis, and dysfunction, were markedly diminished [43–45]. Bone marrow transplantation experiments with apoptosis-associated speck-like adaptor protein-deficient mice revealed that NLRP3 inflammasome activation in bone marrow cells and myocardial resident cells such as cardiomyocytes or cardiac fibroblasts plays a crucial role in myocardial I/R injury [41]. The in vitro experiments revealed that hypoxia/reoxygenation stimulated by NLRP3 inflammasome activation in cardiac fibroblasts and hypoxia/reoxygenation-induced activation was mediated through reactive oxygen species production and potassium efflux [46, 47]. All of these suggested that NLRP3 inflammasome was one of the most important molecular basis for the initial inflammatory response after I/R. Its activation in cardiac fibroblasts was essential for myocardial I/R injury, so it maybe a potential novel therapeutic target for preventing myocardial I/R injury [48–51]. The detailed association between NLRP3 inflammasome and myocardial I/R injury is described in Figure 4.

### 4.3. The Association between NLRP3 Inflammasome and Heart Failure

Inflammation is associated with cardiac remodeling and heart failure, but how it is initiated in response to nonischemic interventions in the absence of cell death is still not very clear. Activation of the NLRP3 inflammasome triggers inflammatory gene expression in cardiomyocytes. These responses could provide signals for macrophage recruitment, fibrosis, and myocardial dysfunction in the heart. These studies suggests targeting early inflammatory responses induced by NLRP3 inflammasome-associated signal can prevent the progression to heart failure [52, 53]. The in vivo study also had shown that Tet2 deficiency in hematopoietic cells is associated with greater cardiac dysfunction in murine models of heart failure as a result of elevated IL-1*β* signaling. Individuals with TET2-mediated clonal hematopoiesis may have greater risk of developing heart failure and respond better to IL-1*β*–NLRP3 inflammasome inhibition [54].

## 5. Discussion and Conclusion

To sum up, these previous results have a number of therapeutic implications. NLRP3 inflammasome could identify a large number of bacteria, viruses, and some endogenous signals that activate caspase-1 and induce production and secretion of IL-1*β* and IL-18. Numerous studies have confirmed that the NLRP3 played a vital role in the atherosclerosis and occurrence of cardiovascular diseases [3, 30, 41]. Based on the activation mode of NLRP3, inhibiting NLRP3 inflammasome activation may have beneficial effects in preventing the damage mediated by the sterile inflammatory response in cardiovascular diseases such as CHD and MI. Preventing pathological NLRP3 inflammasome from activation may provide some insight into the future prevention and treatment of cardiovascular diseases [1, 3, 10]. Currently, the most promising treatments for inhibiting NLRP3 are anti-IL-1, inhibition of caspase-1, and P2X7 receptors antagonist [47–49].

Targeting against the assembly and activity of the NLRP3 inflammasome is a potential and novel therapy for inflammasome-associated diseases, especially for cardiovascular diseases [55–60]. Some studies have indicated that blocking S194 phosphorylation can prevent NLRP3 inflammasome activation. Inhibiting NLRP3 phosphorylation could be an effective treatment for NLRP3-related diseases [61]. In addition, other studies have shown that the NLRP3 NACHT domain molecule may be the target for drug development against cardiovascular diseases. Blocking ATP hydrolysis could inhibit NLRP3 activation and inflammasome formation [62, 63].

Further research on the NLRP3 activation mechanisms and more sophisticated animal experiments as well as clinical trials of molecular targeted agents on NLRP3 are needed to better shed light on the association between NLRP3 inflammasome and cardiovascular diseases, as well as the complicated roles of inflammasome in cardiovascular diseases precisely.
